# Perception and use of flywheel resistance training amongst therapists in sport

**DOI:** 10.3389/fspor.2023.1141431

**Published:** 2023-04-12

**Authors:** Kevin L. de Keijzer, Javier Raya-González, Álvaro López Samanés, Victor Moreno Perez, Marco Beato

**Affiliations:** ^1^ School of Health and Sports Sciences, University of Suffolk, Ipswich, United Kingdom; ^2^ Institute of Health and Wellbeing, University of Suffolk, Ipswich, United Kingdom; ^3^ Faculty of Health Sciences, Universidad Isabel I, Burgos, Spain; ^4^ Faculty of Sport Sciences, University of Extremadura, Cáceres, Spain; ^5^ Exercise Physiology Group, School of Physiotherapy, Faculty of Health Sciences, Universidad Francisco de Vitoria, Madrid, Spain; ^6^ Center for Translational Research in Physiotherapy, Department of Pathology and Surgery, Miguel Hernandez University of Elche, San Joan, Spain

**Keywords:** strength, survey, opinion, team sport, research

## Abstract

Flywheel (isoinertial) resistance training is a valid strength training method that has been incorporated in sport for decades, yet little is known about how therapists working in sport apply flywheel resistance training. We aimed to describe and understand current application and perception of flywheel resistance training amongst therapists working in sport. Seventy- three therapists (13 ± 10 years of experience) started part of this survey with 52 completing the entire electronic questionnaire. Nine multiple choice questions on application and perceptions of flywheel training (prerequisites, use of technology, barriers, and upper- and lower-body exercises) preceded two 6-point Likert scale statements on strength and reduction of injury likelihood. Most therapists (47/73) either used or intended to use flywheel training with their athletes and stated familiarisation would be a priority prior to initiating training. Although more than half suggested they were confident flywheel training could enhance strength (27/52) and muscular prehabilitation outcomes (40/52), many remained unsure. Nonetheless, it appears that therapists would mostly include flywheel training within prehabilitation (40/52) or during the later stages of rehabilitation (37/52). To monitor progress, therapists slightly prefer power (30/52) over velocity outputs, while few would not use them at all. Although therapists would prescribe most exercises - the squat, rotational exercise, and unilateral leg curl would be the most selected. Meanwhile, therapists reported remain most unsure or would avoid prescribing the lateral squat and unilateral hip extension. The biggest perceived barriers to flywheel training are equipment cost/space, evidence, and scheduling. The investigation provides valuable insight into the application and perception of flywheel training amongst therapists working in sport.

## Introduction

A key interest of therapists in sport is the appropriate integration of resistance training methods to reduce likelihood of injury, improve performance, and to rehabilitate athletes from injury (1–3). Flywheel training is a resistance method that has become popular for sport performance and rehabilitation purposes with a variety of healthy and injured athletic populations (4, 5).

Flywheel training requires the user to rotationally accelerate the flywheel during the concentric phase, generating inertial torque that must then be overcome during the eccentric phase (6). If flywheel training is performed appropriately, an eccentric overload (which should be confirmed by mechanical outputs) can be achieved (7). A key benefit of flywheel training is the ability to monitor, adapt, and optimise short- and long-term training with both concentric and eccentric mechanical outputs (8, 9). A variety of bilateral and unilateral flywheel exercises are often prescribed in practice (10–12), although flywheel squats have been the most investigated in the literature (13). The ability to adapt and modify exercises with flywheel devices alongside the maximal resistance provided during both concentric and eccentric phases elicits unique adaptations and are of great interest to practitioners (13). Several examples supporting the efficacy of flywheel training are reported in the literature. For example, 10 weeks of flywheel squat and leg curl training enhanced CMJ height (Hedges *g = *0.60), sprint performance (*g = −*0.84, *moderate*) and reduced injury severity of elite youth soccer players (14). Similarly, 16 sessions of bilateral flywheel leg curl performed over 10 weeks with professional Swedish soccer players significantly improved isokinetic eccentric hamstring strength (*g = *1.14, *moderate*), 30 m sprint (*g = *−0.79, *moderate*), and reduced injury occurrence (15). A narrative review on the benefits of flywheel training also highlights its potential use for rehabilitation and strength development with non-athletic, elderly, and injured populations (5).

While a great deal of research suggests that flywheel training can be an effective resistance training method (10, 16, 17), only recently have investigations into the perceptions and applications of flywheel training begun to emerge (13). It was recently reported that flywheel training frequency prescribed in professional soccer was mostly in line with current guidelines (1–2 sessions per week) (13, 17). Additionally, flywheel training is perceived to be an effective training method to enhance strength (13), which is also in agreement with the literature (4, 18). Nonetheless, differences in perceptions and application of flywheel training amongst soccer practitioners differed from current evidence-based practice and some beliefs were not founded on strong evidence (13). Specifically, a majority of practitioners believed that flywheel training was effective for reducing likelihood of non-contact muscular injuries (13), although only two studies have been performed on the topic (14, 15). Similarly, practitioners' opinions and the current evidence available regarding the enhancement of sport performance with athletes also contrasted (13, 19). Differences between applied practice and scientific evidence have been previously reported in soccer (20). Indeed, discordance between practice and evidence is a common finding amongst coaches and physiotherapists in the literature (21, 22). Surveyed therapists have mostly portrayed negative views on the value and clarity of research (21). This highlights a concerning trend whereby differences exist between therapist application and the underpinning evidence base (1, 2, 13). Indeed, recommendations and best practice proposed within the scientific literature often contrasts what is performed by therapists working in team sports. Such differences are due to the unpredictability associated with the in-season period, time, personnel, or equipment available (1).

Considering therapists have dynamic roles whereby they are often considered to be amongst the most influential members of staff related to injury prevention in elite sport (23), their perception and application of flywheel training is of great interest. This has never been investigated previously and can highlight how therapists in sport are managing the flywheel training process and periodisation with other support staff (i.e*.,* strength and conditioning coaches) (13). Investigating such insights and approaches could bridge the gap between current evidence and practice as well as develop future research directions. Additionally, such an investigation can highlight barriers to evidence-based practice and provide future research directions (1, 21). Therefore, the aim of this study was to describe and understand current application and perception of flywheel-based resistance training amongst therapists working in sport. This study will be the first to contextualise perceptions and applications of therapists working in sport and compare opinions to the current evidence base. This analysis will allow for an appraisal of difficulties that therapists are facing when applying flywheel training and will aid the development of new research avenues. We hypothesised that discrepancies between therapist beliefs and evidence would exist regarding the efficacy of flywheel training for reduction of injury likelihood. Finally, we hypothesise that flywheel devices would mostly be used during late-stage rehabilitation and for reduction of injury likelihood.

Seventy-three therapists (13 ± 10 years. of experience) participated in this study with 52 completing the electronic questionnaire in its entirety. Most participants were physiotherapists (*n* = 54/73) while others were either sports rehabilitators (*n* = 12/73) or sports therapists (*n* = 7/73). Participants were categorized using a framework (24). Specifically, therapists reported to have worked with trained [Tier 2 (*n* = 22/73)], well-trained/national [Tier 3 (*n* = 27/73)], elite/international [Tier 4 (*n* = 16/73)] or world-class [Tier 5 (*n* = 8/73)] athletes. Therapists who worked with Tier 2 & 3 athletes were predominantly based with males (*n* = 14/73) or mixed (*n* = 28/73) populations, while few worked with females only (*n* = 5/73). A similar trend with Tier 4 & 5 athletes was present whereby only one therapist who worked with female populations and a majority worked with male (*n* = 14/73) or mixed athlete populations (*n* = 9/73). Therapists working with a mix of male and female athletes were mostly working in multiple sports (*n* = 23/73), with some working in team-based sports (*n* = 6/73) or athletics (*n* = 4/73), while only one worked in tennis and one in skiing. Therapists working with male athletes predominantly worked in team-based sports (*n* = 23/73), while some worked in multiple sports (*n* = 4/73), athletics (*n* = 3/73), or skiing (*n* = 1/73). Most therapists working with female athletes were also based in team-based sports (*n* = 5/73), while only one worked in athletics. Participants were recruited through social media platforms and authors' professional networks. Sample size was maximized through chain sampling whereby participants were encouraged to invite relevant persons within their networks. The survey was approved by the University of Suffolk (Ipswich, UK) research ethics committee. All participants gave electronic informed consent prior to participation.

### Experimental approach to the problem

Participants completed an online questionnaire through QuestionPro (California, United States). Nine multiple choice questions on application and perceptions of flywheel training (prerequisites, use of technology, barriers, and upper- and lower-body exercises) preceded two 6-point Likert scale statements on strength and reduction of injury likelihood. Videos of included exercises were available (with names and execution) where relevant to standardize exercise terminology and execution between therapists.

### Quantitative analysis

Frequencies were determined for each close-ended question or Likert-type scale response, with many of the responses also presented as frequency plots. All participants were included in the final analysis.

## Results

### Use of flywheel training

A similar number of therapists stated they have often (*n* = 21/73) or sometimes (*n* = 18/73) used flywheel training, while few rarely used it (*n* = 7/73) or do not intend to use it (*n* = 5/73). Interestingly, quite a few therapists reported never having used flywheel training but would like to within their practice (*n* = 22/73).

### Flywheel training prerequisites

**Figure 1:**
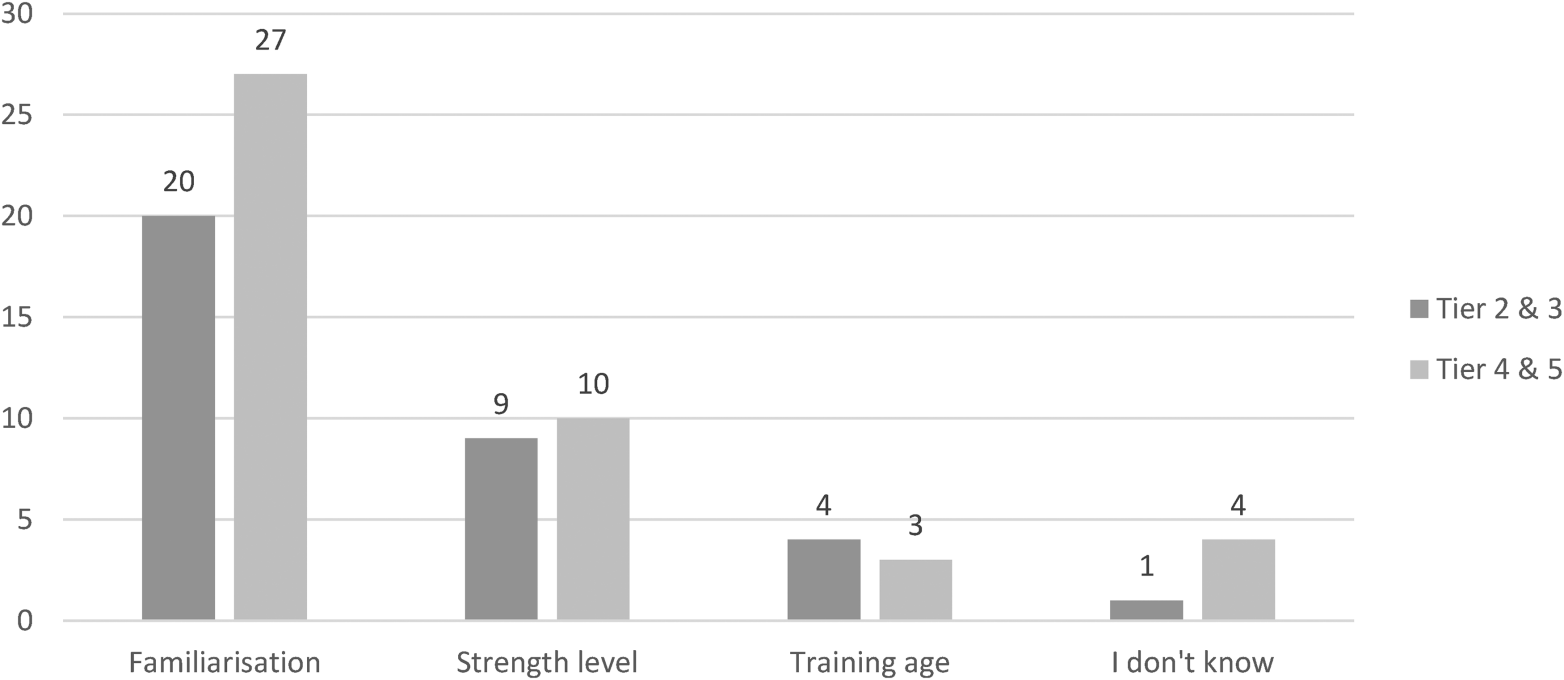
The prerequisites to flywheel training according to therapists with responses tiered by the level of athlete (classified using a framework [23]). *N* = 73.

Most therapists would prioritize movement competency and familiarization (*n* = 47/73) amongst their athletes prior to initiating flywheel training (Figure 1). Following this, the need for sufficient strength (*n* = 19/73) and training age (*n* = 7/73) were perceived as perquisites to flywheel training, while some others remained unsure about what is necessary prior to initiating flywheel training (*n* = 5/73).

### Flywheel training for enhancing strength

**Figure 2:**
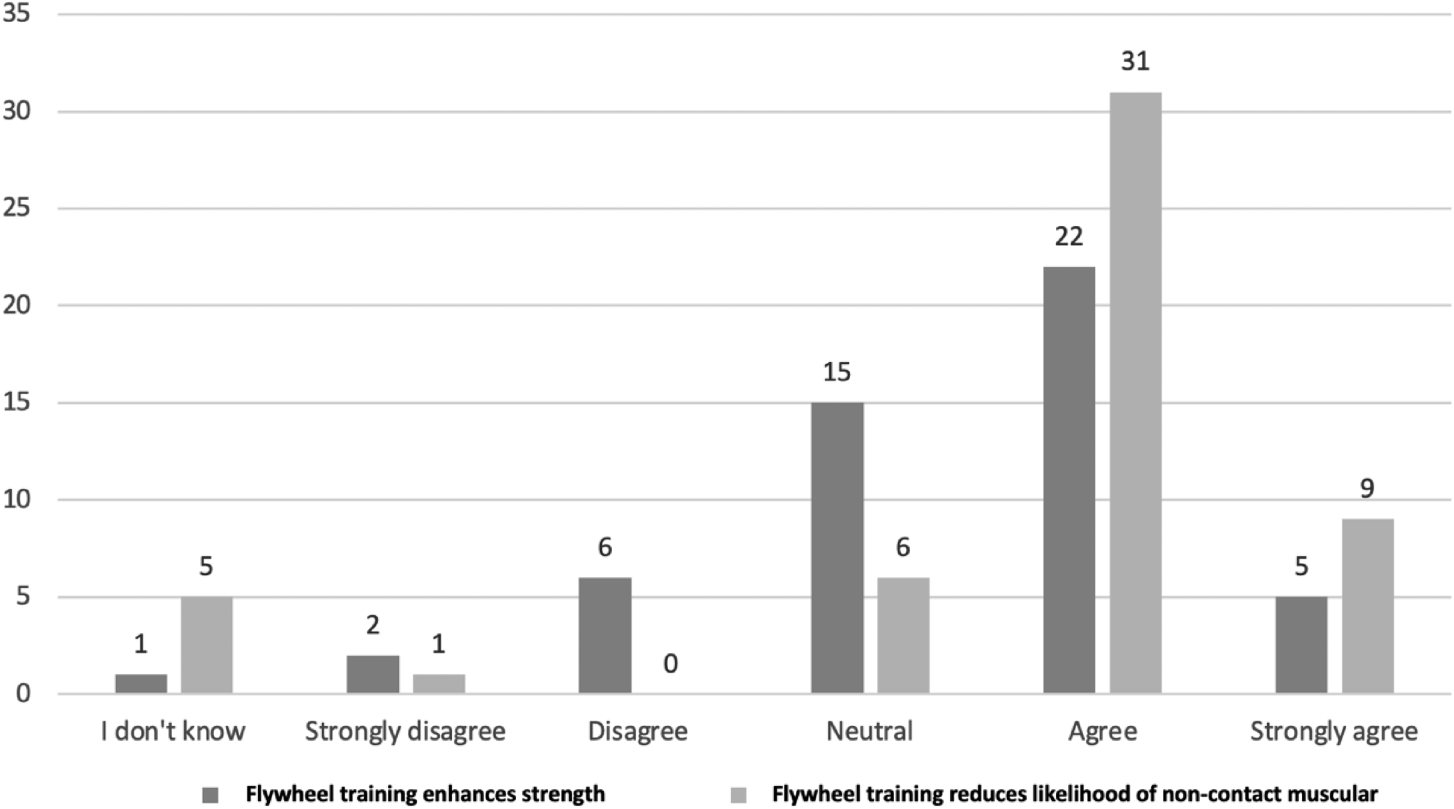
Comparing therapists opinions on the efficacy of flywheel training for reducing likelihood of non-contact muscular injury and enhancing strength. *N* = 52 for each statement.

Although more than half of all therapists agreed or strongly agreed with the statement that flywheel training enhances strength, quite a few were neutral. Interestingly, a few therapists disagreed or strongly disagreed with the statement that flywheel training enhances strength (Figure 2).

### Flywheel training for reducing likelihood of injury and rehabilitation

**Figure 3:**
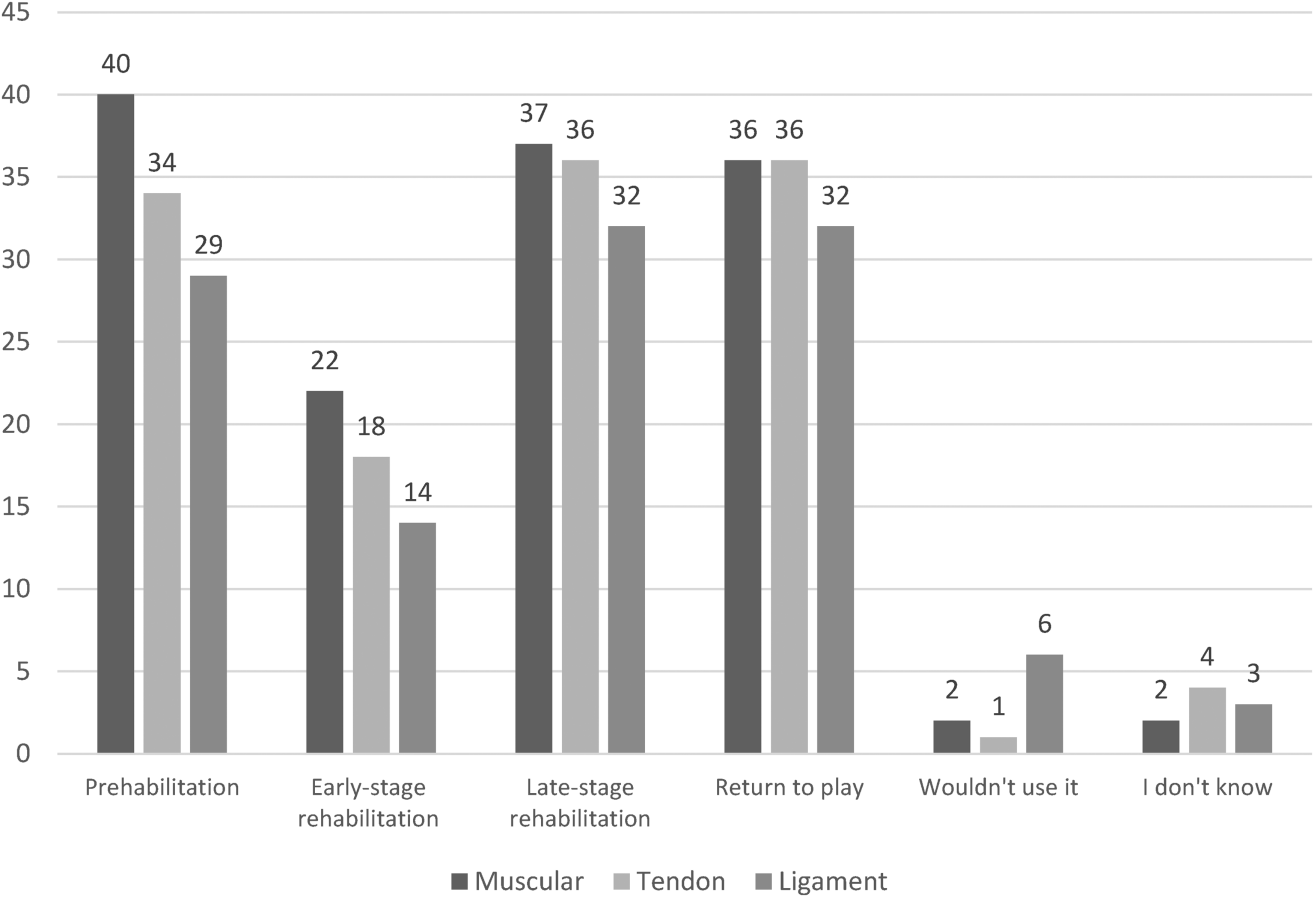
The use of flywheel training for muscular, tendon, and ligament prehabilitation and rehabilitation amongst therapists working in sport. *N* = 52.

Over half of all therapists would utilize flywheel training for reducing non-contact muscular injuries, while less than half would use it for prehabilitation of tendon or ligament injury (Figure 3). In agreement with the previous findings, most therapists agree that flywheel training can reduce non-contact muscular injury likelihood, while few strongly agreed (Figure 2).

Therapists would mostly use flywheel training within late-stage rehabilitation and return to play/re-integration in comparison to early-stage rehabilitation (Figure 3). More uncertainty remains regarding the use of flywheel training for prehabiliation (as part of injury prevention) and rehabilitation of tendon and ligament injury in comparison to muscular injury (Figure 3).

### Mechanical outputs used during flywheel training

Most therapists prefer peak (*n* = 30/52) or average (*n* = 29/52) power over peak (*n* = 24/52) or average (*n* = 25/52) speed/velocity as a metric to monitor or program flywheel training. Only few (*n* = 6/52) stated they would not use metrics with the same number of therapists (*n* = 6/52) would use other metrics (Eccentric power, impulse, balance).

### Flywheel exercise selection

**Figure 4:**
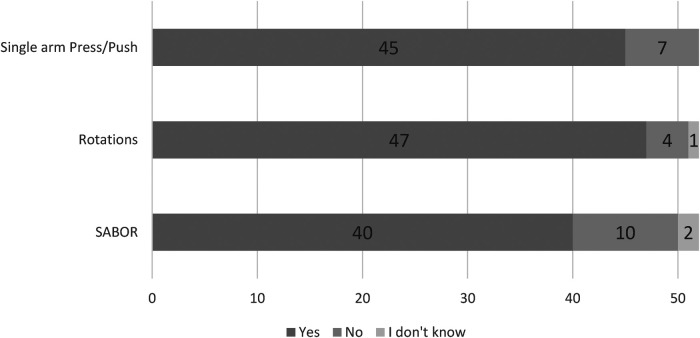
The use of upper body flywheel exercises amongst therapists working in sport. *N* = 52.

**Figure 5:**
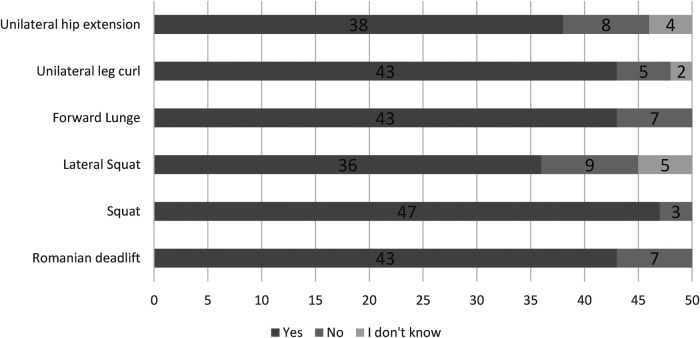
The use of lower body flywheel exercises amongst therapists working in sport. *N* = 50.

Although most therapists would utilize the single arm bent over row (SABOR), almost all would use the single arm press or rotational exercises (Figure 4). The lower-body exercises most favored by therapists are the squat, followed by the Romanian deadlift, forward lunge, and unilateral leg curl (Figure 5). For the lower body, although a majority of therapists would prescribe lateral squats and unilateral hip extensions, some also remained unsure or would not prescribe those exercises (Figure 5).

### Barriers to flywheel training

The biggest barriers to the use of flywheel training amongst therapists are equipment cost or space (*n* = 31/52). Although some therapists perceive there to be no barriers (*n* = 10/52), others perceive evidence and knowledge (*n* = 17/52), scheduling (*n* = 10/52), bias and culture (*n* = 7/52), or safety (*n* = 3/52) to be barriers to use of flywheel training.

## Discussion

The aims of the study were to compare and understand application and perception of flywheel-based resistance training amongst therapists working in sport. Important differences between the perceived benefit and actual evidence for using flywheel training to reduce injury likelihood exist. In agreement with our hypothesis, most therapists utilised flywheel training predominantly for reduction of injury likelihood and re-incorporate flywheel training during the latter stages of rehabilitation.

### Prerequisites to flywheel training

Most therapists would prioritize movement competency and familiarization (*n* = 47/73) amongst their athletes prior to initiating flywheel training. The present findings agree with previous findings in professional soccer whereby familiarization was mostly perceived to be necessary to optimize training (13). The perceived need for familiarization is supported by current guidelines (25), although a variety of approaches to obtain movement competency and familiarize athletes have been reported in the literature (13). The combination of objective mechanical outputs (i.e., velocity) (8), qualitative feedback, and sufficient athlete confidence are likely to optimize familiarization and movement competency with flywheel training. Interestingly, other therapists believe that strength (*n* = 19/73) and training age (*n* = 7/73) are prerequisites to flywheel training, although little is known about the impact of such characteristics on efficacy of flywheel training (25). The need for sufficient strength or training age prior to introduction of flywheel training may be particularly relevant with youth populations or athletes rehabilitating from surgery or long-term injuries (26–28). It is likely that some (*n* = 5/73) therapists remained unsure about what is necessary prior to initiating flywheel training because quite a few (*n* = 22/73) of the investigated therapists had not used flywheel training previously.

### Flywheel training for enhancing strength

Most therapists agree that flywheel training can enhance strength (*n* = 27/52), which is in agreement with previous reports that flywheel training is perceived to effectively enhance strength of professional soccer players (13). An umbrella review specifically on the effects of flywheel training on strength parameters supports the present and previous findings (4). Although the need for strength and power training during rehabilitation is evident (3), quite a few therapists stated they were either neutral (*n* = 15/52) or disagree (*n* = 8/52) with the statement that flywheel training was effective for enhancing strength (Figure 2). It is possible that a greater amount of research specific to soccer (19) encouraged greater confidence for strength enhancement amongst practitioners working in soccer (13). Meanwhile, the limited evidence within the realm of rehabilitation and sports therapy does not provide as much confidence for use of flywheel training and may explain the present findings (5, 27, 28).

### Exercise selection

Similar to previous findings in elite soccer (13), the flywheel squat would be the most used flywheel exercise amongst therapists in sport (Figure 5). A previous survey amongst practitioners working in elite sport reported a similar trend, whereby a majority would also prescribe variations of eccentric squats more so than other typologies of eccentric exercises (22). The greater evidence base available for bilateral flywheel squats (14, 16, 29–31) in comparison to lateral squats (10, 32) or lunges (12) may influence the perceptions of therapists working in sport. Indeed, one of the bigger reported barriers to application of flywheel training in the present study was evidence and knowledge (*n* = 17/52). In contrast to the theory that a greater evidence base guides exercise selection, a great deal of therapists would prescribe the Romanian deadlift, unilateral leg curl, and upper body rotational exercises (Figures 4, 5). Therapists would utilise such exercises even though very limited evidence is available regarding such lower body flywheel exercises (33–35). The present study is the first to investigate the use of upper body flywheel exercises amongst therapists, reporting that almost all therapists would use rotational exercises while fewer would consider unilateral pull and push exercises (Figure 4). Three weekly sessions over a 12-week period of unilateral pull and rotational flywheel exercises enhanced shoulder isometric strength and range of motion in comparison to a control condition (which also included strength training) with youth tennis athletes (36). Another bi-weekly program of either flywheel or pneumatic resistance training similarly enhanced isokinetic shoulder strength, hypertrophy, and throwing speed outcomes with professional hand ball athletes (37). Although the flywheel literature is limited, it is likely that therapists rely on barbell-based (upper body or posterior chain) exercise literature that has been commonplace and easily accessible (3, 38, 39). It is also likely that if evidence is not available for a specific exercise then other variations (i.e., bilateral instead of unilateral leg curl) are used to form opinions on specific flywheel exercises (14, 15, 35). For example, although no longitudinal studies have been performed on the effects of unilateral leg curl, a plethora of evidence supports the use of open kinetic chain exercises and may therefore justify inclusion of similar open kinetic chain exercises (14, 15, 35, 40, 41). It is likely that although limited evidence supporting the use of specific unilateral, upper body exercises or flywheel training with certain populations (i.e., females) is available (42), therapists simply draw conclusions based on what they are able to access (43). Future investigations should study the effects of manipulating training variables (moment of inertia, volume, exercises) with specific populations of interest to increase the efficacy of flywheel training programs (17). Indeed, although the biggest barriers to the use of flywheel training is equipment cost or space (*n* = 31/52), further high-quality investigations may reduce the impact of other barriers (scheduling, bias and culture, or safety) on the application of flywheel training (17).

### Flywheel training for prehabilitation and rehabilitation

Strength training is considered a key factor related for reducing likelihood of injury (23). In agreement with this, most therapists believe that flywheel training is effective for reducing likelihood of non-contact muscular injury (Figure 2). The use of flywheel training to reduce likelihood of muscular injury has been previously reported amongst practitioners working in elite soccer and is supported by few investigations that have reported beneficial outcomes (13–15). Meanwhile, the limited evidence supporting the use of flywheel training for prehabilitation of tendon or ligament injuries is reflected in the present study with fewer therapists utilizing it for such purposes (Figure 3).

The re-integration of flywheel training during rehabilitation by therapists in the present study is supported by the enhanced neuromuscular capacity, musculotendinous stiffness, and connective tissue strength associated with maximal strength training during rehabilitation (3). The present findings suggest therapists would re-integrate intense eccentric training (i.e., flywheel training) during late-stage rather than early-stage rehabilitation (Figure 3), in line with traditional recommendations of progressive rehabilitation guidelines (3). As expected, a greater number of therapists would re-introduce flywheel training sooner when rehabilitating muscular injuries (*n* = 22/52) in comparison to tendon (*n* = 18/52) or ligament (*n* = 14/52) injuries. Such views are supported by the specific characteristics and advantages of flywheel training that may simultaneously reduce likelihood of re-injury and enhance performance (38). Although published trials involving re-introduction of flywheel training within the early phase of rehabilitation are unavailable, evidence supporting the use of lengthening exercises earlier within rehabilitation with progressively increased eccentric load are promising (44, 45). Specifically, training with an eccentric emphasis significantly reduced time to return [28 (8–58) days] in comparison to a conventional training protocol [51 (12–94) days] (45), supporting the use of flywheel training within rehabilitation. In support of the approach utilized by some therapists in the present investigation, re-introduction of lengthening exercises earlier in the rehabilitation of muscular injuries also quickened return to sport (23 days) compared to a typical “delayed lengthening” protocol (33 days) without negatively impacting re-injury rates between groups (44).

Although the aforementioned evidence suggests benefit of earlier re-introduction of flywheel training after muscular injury, the re-integration of flywheel training in the later stages of tendon (*n* = 36/52) and ligament (*n* = 32/52) rehabilitation is justified (Figure 3) (5, 28). The high mechanical load that is usually obtained with flywheel training provides a unique stimulus and can enhance strength, hypertrophy and tendon adaptations but may not be appropriate during the early stages of rehabilitation (4, 5, 28). Although limited evidence for treatment of pathological tendons of athletic populations with flywheel training exists, investigations lasting 6–12 weeks have shown enhanced pain and function related outcomes amongst young and athletic populations (46, 47). In support of the use of flywheel training for tendon rehabilitation amongst therapists, a recent scoping review reports that the four studies on flywheel training for tendon rehabilitation have all enhanced patellar tendon outcomes (i.e., tendon thickness) (28). Not as many therapists are utilizing flywheel training to rehabilitate ligament injuries as they are for muscular or tendon injuries (Figure 3). Therapists cannot utilize flywheel specific evidence but may likely rely on reviews and guidelines previously developed with traditional resistance training since a plethora of evidence suggests that flywheel training may be used as an alternative strength training method to traditional resistance training methods (3, 38).

## Limitations and future directions

This study is not without limitations. The present investigation could be limited by various types of bias (e.g., affecting respondent participation and responses given) and is likely not to be representative of all therapists working in sport. Specifically, practitioners who are more confident or well versed with flywheel training may have been more likely to participate. Nonetheless, the present study increases awareness of perceived limitations and how flywheel training is used amongst a population of experienced therapists working with a variety of athletes. The present study highlights future investigations should aim to also provide further information on the use of metrics (such as power and speed/velocity) to guide training and rehabilitation. Among the barriers to flywheel training, it is likely that athlete and coach buy- in or scheduling impact the integration of flywheel training within the prehabilitation programs of elite and world class team sport athletes - although this must be confirmed in future investigations. Although further research specific to flywheel training is necessary to support the current beliefs that flywheel training is effective for rehabilitation of muscular, tendon, and especially ligament injuries, therapists can continue to utilize flywheel training within a holistic plan (involving neuromuscular and dynamic balance training) to accelerate on-field training and rehabilitation.

## Conclusions

This survey is the first to provide valuable insight into the application and perception of flywheel training amongst therapists working in sport. Most therapists use or would like to use flywheel resistance technology during some phases of the rehabilitation process with their athletes. They stated, that based on their experience, appropriate familiarisation is a priority when flywheel technology is implemented. More than half of them suggested they were confident flywheel training could enhance strength and muscular prehabilitation outcomes, however, many remained unsure about the benefits of flywheel technology. This underlines that some therapists are not fully aware of the latest scientific evidence or believe flywheel training is ineffective. Nonetheless, it appears that therapists would mostly include flywheel training within prehabilitation and during the later stages of rehabilitation. Additionally, from a training load monitoring point of view, therapists use both power and velocity outputs, although several do not use these parameters. Although therapists would prescribe most exercises - the squat, rotational core exercise, and unilateral leg curl would be the most selected. Meanwhile more therapists reported being unsure or avoiding prescription of the lateral squat and unilateral hip extension. Finally, the biggest perceived barriers to flywheel training are equipment cost/space, evidence, and available time for its implementation. We believe that there is a gap in knowledge between practitioners (working industry) and researchers (academia), and this should be reduced with education programmes and tailored courses for practitioners that could facilitate the implementation of this technology in sports and physiotherapy.

## Data Availability

The raw data supporting the conclusions of this article will be made available by the authors, without undue reservation.

## References

[1] Meurer MC Silva MF Baroni BM. Strategies for injury prevention in Brazilian football: perceptions of physiotherapists and practices of premier league teams. Phys Ther Sport. (2017) 28:1–8. 10.1016/j.ptsp.2017.07.004 28886473

[2] McCall A Carling C Davison M Nedelec M Le Gall F Berthoin S Injury risk factors, screening tests and preventative strategies: a systematic review of the evidence that underpins the perceptions and practices of 44 football (soccer) teams from various premier leagues. Br J Sports Med. (2015) 49:583–9. 10.1136/bjsports-2014-094104 25576530 PMC4413799

[3] Maestroni L Read P Bishop C Turner A. Strength and power training in rehabilitation: underpinning principles and practical strategies to return athletes to high performance. Sport Med. (2020) 50:239–52. 10.1007/s40279-019-01195-6 31559567

[4] de Keijzer KL Gonzalez JR Beato M. The effect of flywheel training on strength and physical capacities in sporting and healthy populations: an umbrella review. PLoS One. (2022) 17:e0264375. 10.1371/journal.pone.0264375 35213634 PMC8880830

[5] Tesch PA Fernandez-Gonzalo R Lundberg TR. Clinical applications of iso-inertial, eccentric-overload (YoYo™) resistance exercise. Front Physiol. (2017) 8:241. 10.3389/fphys.2017.00241 28496410 PMC5406462

[6] Berg HE Tesch A. A gravity-independent ergometer to be used for resistance training in space. Aviat Space Environ Med. (1994) 65(8):752–6. PMID: . 7980338

[7] Muñoz-López A de Souza Fonseca F Ramírez-Campillo R Gantois P Javier Nuñez F Nakamura F. The use of real-time monitoring during flywheel resistance training programmes: how can we measure eccentric overload? A systematic review and meta-analysis. Biol Sport. (2021) 38(4):639–52. 10.5114/biolsport.2021.101602 34937974 PMC8670814

[8] Martín-Rivera F Beato M, Alepuz-Moner V, Maroto-Izquierdo S. Use of concentric linear velocity to monitor flywheel exercise load. Front Physiol. (2022) 13:961572. 10.3389/fphys.2022.961572 36035469 PMC9412162

[9] Maroto-Izquierdo S Raya-González J Hernández-Davó JL Beato M. Load quantification and testing using flywheel devices in sports. Front Physiol. (2021) 12:739399. 10.3389/fphys.2021.739399 34777007 PMC8587883

[10] Raya-González J Castillo D de Keijzer KL Beato M. The effect of a weekly flywheel resistance training session on elite U-16 soccer players’ physical performance during the competitive season. A randomized controlled trial. Res Sport Med. (2021) 29(6):571–85. 10.1080/15438627.2020.1870978 33401975

[11] Sagelv EH Pedersen S Nilsen LPR Casolo A Welde B Randers MB Flywheel squats versus free weight high load squats for improving high velocity movements in football. A randomized controlled trial. BMC Sports Sci Med Rehabil. (2020) 12:61. 10.1186/s13102-020-00210-y 33024564 PMC7532637

[12] Tous-Fajardo J Gonzalo-Skok O Arjol-Serrano JL Tesch P. Enhancing change-of-direction speed in soccer players by functional inertial eccentric overload and vibration training. Int J Sports Physiol Perform. (2016) 11:66–73. 10.1123/ijspp.2015-0010 25942419

[13] de Keijzer K McErlain-Naylor SA Brownlee ET Raya-González J Beato M. Perception and application of flywheel training by professional soccer practitioners. Biol Sport. (2022) 39(4):809–17. 10.5114/biolsport.2022.109457 36247955 PMC9536362

[14] de Hoyo M Pozzo M Sañudo B Carrasco L Gonzalo-Skok O Domínguez-Cobo S Effects of a 10-week in-season eccentric-overload training program on muscle-injury prevention and performance in junior elite soccer players. Int J Sports Physiol Perform. (2015) 10:46–52. 10.1123/ijspp.2013-0547 24910951

[15] Askling C Karlsson J Thorstensson A. Hamstring injury occurrence in elite soccer players after preseason strength training with eccentric overload. Scand J Med Sci Sports. (2003) 13:244–50. 10.1034/j.1600-0838.2003.00312.x 12859607

[16] Coratella G Beato M Cè E Scurati R Milanese C Schena F Effects of in-season enhanced negative work-based vs traditional weight training on change of direction and hamstrings-to-quadriceps ratio in soccer players. Biol Sport. (2019) 36:241–8. 10.5114/biolsport.2019.87045 31624418 PMC6786325

[17] Beato M Maroto-Izquierdo S Hernández-Davó JL Raya-González J. Flywheel training periodization in team sports. Front Physiol. (2021) 12:732802. 10.3389/fphys.2021.732802 34819871 PMC8606557

[18] Raya-González J Castillo D de Keijzer KL Beato M. Considerations to optimize strength and muscle mass gains through flywheel resistance devices: a narrative review. Strength Cond J. (2023) 45(1):111–21. 10.1519/SSC.0000000000000732

[19] Allen WJC de Keijzer KL Raya-González J Castillo D Coratella G Beato M. Chronic effects of flywheel training on physical capacities in soccer players: a systematic review. Res Sport Med. (2021):1–21. 10.1080/15438627.2021.1958813 34315310

[20] McCall A Carling C Nedelec M Davison M Le Gall F Berthoin S Risk factors, testing and preventative strategies for non-contact injuries in professional football: current perceptions and practices of 44 teams from various premier leagues. Br J Sports Med. (2014) 48:1352–7. 10.1136/bjsports-2014-093439 24837243

[21] Grimmer-Somers K Lekkas P Nyland L Young A Kumar S. Perspectives on research evidence and clinical practice: a survey of Australian physiotherapists. Physiother Res Int. (2007) 12:147–61. 10.1002/pri.363 17624895

[22] Harden M Bruce C Wolf A Hicks KM Howatson G. Exploring the practical knowledge of eccentric resistance training in high-performance strength and conditioning practitioners. Int J Sports Sci Coach. (2020) 15:41–52. 10.1177/1747954119891154

[23] Gregson W Carling C Gualtieri A O’Brien J Reilly P Tavares F A survey of organizational structure and operational practices of elite youth football academies and national federations from around the world: a performance and medical perspective. Front Sport Act Living. (2022) 4:1031721. 10.3389/fspor.2022.1031721 PMC9727309 36506723

[24] McKay AKA Stellingwerff T Smith ES Martin DT Mujika I Goosey-Tolfrey VL Defining training and performance caliber: a participant classification framework. Int J Sports Physiol Perform. (2022) 17:317–31. 10.1123/ijspp.2021-0451 34965513

[25] Beato M Dello Iacono A. Implementing flywheel (isoinertial) exercise in strength training: current evidence, practical recommendations, and future directions. Front Physiol. (2020) 11:569. 10.3389/fphys.2020.00569 32581845 PMC7283738

[26] Drury B Ratel S Clark CCT Fernandes JFT Moran J Behm DG. Eccentric resistance training in youth: perspectives for long-term athletic development. J Funct Morphol Kinesiol. (2019) 4:70. 10.3390/jfmk4040070 33467385 PMC7739302

[27] Wonders J. Flywheel training in musculoskeletal rehabilitation: a clinical commentary. Int J Sports Phys Ther. (2019) 14:994–1000. 10.26603/ijspt20190994 31803531 PMC6878857

[28] Burton I McCormack A. Inertial flywheel resistance training in tendinopathy rehabilitation: a scoping review. Int J Sports Phys Ther. (2022) 17(5):775–86. 10.26603/001c.36437 35949372 PMC9340832

[29] Fernandez-Gonzalo R Lundberg TR Alvarez-Alvarez L de Paz JA. Muscle damage responses and adaptations to eccentric-overload resistance exercise in men and women. Eur J Appl Physiol. (2014) 114:1075–84. 10.1007/s00421-014-2836-7 24519446

[30] Sabido R Hernández-Davó JL Botella J Navarro A Tous-Fajardo J. Effects of adding a weekly eccentric-overload training session on strength and athletic performance in team-handball players. Eur J Sport Sci. (2017) 17:530–8. 10.1080/17461391.2017.1282046 28152673

[31] Gual G Fort-Vanmeerhaeghe A Romero-Rodríguez D Tesch PA. Effects of in-season inertial resistance training with eccentric overload in a sports population at risk for patellar tendinopathy. J Strength Cond Res. (2016) 30:1834–42. 10.1519/JSC.0000000000001286 26670989

[32] Madruga-Parera M Bishop C Fort-vanmeerhaeghe A Beato M Gonzalo-skok O Romero-rodr D. Effects of 8 weeks of isoinertial vs. cable- resistance training on motor skills performance and interlimb asymmetries. J Strength Cond Res. (2022) 36(5):1200–8. 10.1519/JSC.0000000000003594 32379241

[33] de Keijzer KL McErlain-Naylor SA Beato M. The effect of flywheel inertia on peak power and its inter-session reliability during two unilateral hamstring exercises: leg curl and hip extension. Front Sport Act Living. (2022) 4:898649. 10.3389/fspor.2022.898649 PMC9226424 35755611

[34] Brien J Browne D Earls D Lodge C. The effects of varying inertial loadings on power variables in the flywheel Romanian deadlift exercise. Biol Sport. (2022) 39:499–503. 10.5114/biolsport.2022.106159 35959332 PMC9331332

[35] Presland JD Opar DA Williams MD Hickey JT Maniar N Lee Dow C Hamstring strength and architectural adaptations following inertial flywheel resistance training. J Sci Med Sport. (2020) 23:1093–9. 10.1016/j.jsams.2020.04.007 32461050

[36] Fernandez-Fernandez J Moreno-Perez V Cools A Nakamura FY Teixeira AS Ellenbecker T The effects of a compensatory training program adding an isoinertial device in the shoulder function on young tennis players. J Strength Cond Res. (2022). 10.1519/JSC.0000000000004374 36399152

[37] Maroto-Izquierdo S McBride JM Gonzalez-Diez N García-López D González- Gallego J de Paz JA. Comparison of flywheel and pneumatic training on hypertrophy, strength, and power in professional handball players. Res Q Exerc Sport. (2022) 93:1–15. 10.1080/02701367.2020.1762836 32669052

[38] Beato M Maroto-Izquierdo S Turner AN Bishop C. Implementing strength training strategies for injury prevention in soccer: scientific rationale and methodological recommendations. Int J Sports Physiol Perform. (2021) 16(3):456–61. 10.1123/ijspp.2020-0862 33503589

[39] Suchomel TJ Nimphius S Bellon CR Stone MH. The importance of muscular strength: training considerations. Sport Med. (2018) 48:765–85. 10.1007/s40279-018-0862-z 29372481

[40] Lundberg TR García-Gutiérrez MT Mandić M Lilja M Fernandez-Gonzalo R. Regional and muscle-specific adaptations in knee extensor hypertrophy using flywheel versus conventional weight-stack resistance exercise. Appl Physiol Nutr Metab. (2019) 44:827–33. 10.1139/apnm-2018-0774 30620623

[41] de Hoyo M Sañudo B Carrasco L Mateo-Cortes J Domínguez-Cobo S Fernandes O Effects of 10-week eccentric overload training on kinetic parameters during change of direction in football players. J Sports Sci. (2016) 34:1380–7. 10.1080/02640414.2016.1157624 26963941

[42] Raya-González J de Keijzer KL Bishop C Beato M. Effects of flywheel training on strength-related variables in female populations. A systematic review. Res Sport Med. (2022) 30(4):353–70. 10.1080/15438627.2020.1870977 33401963

[43] Fullagar HHK Harper LD Govus A McCunn R Eisenmann J McCall A. Practitioner perceptions of evidence-based practice in elite sport in the United States of America. J Strength Cond Res. (2019) 33:2897–904. 10.1519/JSC.0000000000003348 31453942

[44] Vermeulen R Whiteley R van der Made AD van Dyk N Almusa E Geertsema C Early versus delayed lengthening exercises for acute hamstring injury in male athletes: a randomised controlled clinical trial. Br J Sports Med. (2022) 56:792–800. 10.1136/bjsports-2020-103405 35338036 PMC9252858

[45] Askling C Tengvar M Thorstensson A. Acute hamstring injuries in Swedish elite football: a prospective randomised controlled clinical trial comparing two rehabilitation protocols. Br J Sports Med. (2013) 47:986–91. 10.1136/bjsports-2013-092676 23536466

[46] Romero-Rodriguez D Gual G Tesch PA. Efficacy of an inertial resistance training paradigm in the treatment of patellar tendinopathy in athletes: a case-series study. Phys Ther Sport. (2011) 12(1):43–8. 10.1016/j.ptsp.2010.10.003 21256449

[47] Ruffino D Malliaras P Marchegiani S Campana V. Inertial flywheel vs heavy slow resistance training among athletes with patellar tendinopathy: a randomised trial. Phys Ther Sport. (2021) 52:30–7. 10.1016/j.ptsp.2021.08.002 34384941

